# Tools for microbial single-cell genomics for obtaining uncultured microbial genomes

**DOI:** 10.1007/s12551-023-01124-y

**Published:** 2023-09-08

**Authors:** Masahito Hosokawa, Yohei Nishikawa

**Affiliations:** 1 Department of Life Science and Medical Bioscience, Waseda University, 2-2 Wakamatsu-Cho, Shinjuku-Ku, Tokyo, 162-8480 Japan; 2 Computational Bio Big-Data Open Innovation Laboratory, National Institute of Advanced Industrial Science and Technology, 3-4-1 Okubo, Shinjuku-Ku, Tokyo, 169-8555 Japan; 3 Research Organization for Nano and Life Innovation, Waseda University, 513 Wasedatsurumaki-Cho, Shinjuku-Ku, Tokyo, 162-0041 Japan; 4 Institute for Advanced Research of Biosystem Dynamics, Waseda Research Institute for Science and Engineering, 3-4-1 Okubo, Shinjuku-Ku, Tokyo, 169-8555 Japan; 5 bitBiome, Inc., 513 Wasedatsurumaki-Cho, Shinjuku-Ku, Tokyo, 162-0041 Japan

**Keywords:** Microbiome, Single-cell genomics, Metagenomics, Sequencing, Microfluidics

## Abstract

The advent of next-generation sequencing technologies has facilitated the acquisition of large amounts of DNA sequence data at a relatively low cost, leading to numerous breakthroughs in decoding microbial genomes. Among the various genome sequencing activities, metagenomic analysis, which entails the direct analysis of uncultured microbial DNA, has had a profound impact on microbiome research and has emerged as an indispensable technology in this field. Despite its valuable contributions, metagenomic analysis is a “bulk analysis” technique that analyzes samples containing a wide diversity of microbes, such as bacteria, yielding information that is averaged across the entire microbial population. In order to gain a deeper understanding of the heterogeneous nature of the microbial world, there is a growing need for single-cell analysis, similar to its use in human cell biology. With this paradigm shift in mind, comprehensive single-cell genomics technology has become a much-anticipated innovation that is now poised to revolutionize microbiome research. It has the potential to enable the discovery of differences at the strain level and to facilitate a more comprehensive examination of microbial ecosystems. In this review, we summarize the current state-of-the-art in microbial single-cell genomics, highlighting the potential impact of this technology on our understanding of the microbial world. The successful implementation of this technology is expected to have a profound impact in the field, leading to new discoveries and insights into the diversity and evolution of microbes.

With the advancements in DNA sequencing and bioinformatics, single-cell genomics has experienced rapid technological progress. The first report of single-cell RNA sequencing (scRNA-seq) was published in 2009 (Tang et al. 2009), and since then, various methods have been developed that allow the analysis of several hundred thousand cells (Svensson et al. 2018). The measurement targets have expanded from RNA to genomes, and single-cell multi-omics analysis, including proteins and metabolites, is now becoming a reality.

In most cases, “single cell” refers to mammalian cells, such as those from humans and mice. However, most single-cell genomics techniques are not applicable to the analysis of environmental microbes such as bacteria. This is because the amount of DNA contained in a single bacterium is about 1/1000th of that in a mammalian cell, and the effect of contaminating DNA on the reaction environment is so severe that sequencing analysis from a single microbial cell requires an extremely precise genome amplification process. Nevertheless, there is a growing trend in microbial research to discuss the state of biological populations on a single-cell basis (Blainey 2013; Gawad et al. 2016; Woyke et al. 2017; Blattman et al. 2020; Kuchina et al. 2020). In this review, we present the background, challenges, and recent technological trends in bacterial single-cell genomics.

As mentioned above, scRNA-seq is the most widely used type of single-cell genomics, but in the case of microbial single-cell genomics, genomic DNA rather than RNA is the primary target of analysis. This is because reference genome sequences are often lacking for microbes, and single-cell genomics techniques are used to determine the genomes of unknown microbes. Even bacteria that are commonly handled in laboratories, such as *Escherichia coli*, have a variety of phenotypes, including pathogenic and commensal strains. Therefore, it is essential to explore the factors that lead to the phenotype of each bacterial strain from whole genome analysis to understand the diversity within a species. In addition, since many environmental microbes are unculturable, it is necessary to develop methods for obtaining microbial genomes without isolating and culturing them.

## Approaches for obtaining uncultured microbial genomes

**Fig. 1:**
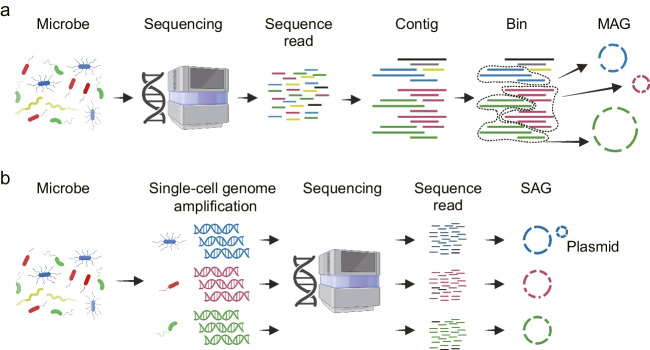
Metagenomics (**a**) and single-cell genomics (**b**): two approaches for obtaining genomes of uncultured microbes

Metagenomics and single-cell genomics are two approaches for obtaining the genomes of uncultured microbes without the need for isolation culture (Bowers et al. 2017). Metagenomics involves the direct sequencing of DNA extracted from microbial populations, and the genomic sequence of each microbe is subsequently classified *in silico* from the mixture of fragmented sequences (Fig. 1a). Single-cell genomics entails the physical cell isolation and amplification of DNA from individual microbes, followed by sequencing (Fig. 1b). The draft genomes obtained from these sequencing efforts are referred to as metagenome-assembled genomes (MAGs) and single-amplified genomes (SAGs), respectively. MAGs and SAGs are acquired through specialized procedures that differ from those used for isolated microbes and, as such, often contain sequence errors and be incomplete. To assess the quality of MAGs and SAGs, a classification guideline has been proposed (Bowers et al. 2017), dividing them into four categories: finished, high quality, medium quality, and low quality. This classification is based on criteria such as the degree of fragmentation of the genome sequence (contigs), the recovery of rRNA genes, the number of tRNA genes, and the estimated completeness and contamination of the genome. In most cases, high- and medium-quality MAGs or SAGs are used to interpret microbial functions. The completeness and contamination are evaluated based on the sufficiency or duplication of single-copy marker genes, and tools such as CheckM are used for this estimation (Parks et al. 2015).

In metagenomics, DNA fragments derived from a diverse array of microbes are sequenced and assembled computationally to generate contigs as consensus sequences. As the metagenomic contig set is a mixture of genome sequences from various microbes, binning is performed to separate the contigs into groups to recover the genome of each individual microbe (Sangwan et al. 2016). The recovery of individual microbial genomes relies on the accurate binning of the contigs into sequence groups called bins. This binning procedure compares contigs based on sequence characteristics such as GC content, tetranucleotide frequency, and sequence coverages (Breitwieser et al. 2019; Yang et al. 2021). Of the bins thus obtained and curated, those that meet standards of genome quality become MAGs. With the advent of next-generation sequencing technologies, a significant number of MAGs have been registered in public genome databases (Parks et al. 2017; Pasolli et al. 2020; Nayfach et al. 2021), with more than 60% of the total 65,703 genomes in the Genome taxonomy database being MAG origin (as of April 2022) (Parks et al. 2022).

Currently, a variety of binning tools have been developed, each competing for classification accuracy in MAGs (Sczyrba et al. 2017; Wu et al. 2022). It is worth noting that different tools output different MAGs from the same raw metagenomic sequence data. As there is no definitive method for evaluating the validity of MAGs obtained from metagenomes in actual environmental samples, they are often utilized for analysis as long as they are not low-quality MAG. However, upon closer examination, MAGs often contain various genome sequences from different microbes and output as chimeric sequences (Shaiber and Eren 2019; Chen et al. 2020; Arikawa et al. 2021). Genome assemblies frequently generate errors with similar sequences across species, such as rRNA genes (Zhang et al. 2016). Binning also poses challenges in accurately sorting exogenous genes, such as plasmids and phages (Maguire et al. 2020), into bins. Ribosomal protein genes are also likely to be absent from MAG (Mise and Iwasaki 2022). It has been reported that only about 7% of MAGs obtained from short-read sequencers contain 16S rRNA genes (Hiseni et al. 2021), which makes it challenging to correlate MAGs with the versatile 16S rRNA gene-based microbiome studies. In the future, long-read sequencers are expected to improve the accuracy of sequence assembly and facilitate the acquisition of near full-length MAGs (Bickhart et al. 2022), and the use of long-read sequencing technologies with PacBio (Feng et al. 2022; Kim et al. 2022) and Oxford Nanopore Technologies (Moss et al. 2020; Ciuffreda et al. 2021; Liu et al. 2022; Orellana et al. 2023) is becoming widespread. However, it has also been reported that the classification of contigs within the same microbial species or genus is difficult even with long-read sequencing when the accuracy of base calling is low and that the classification of genome sequences of specific gut bacteria with many closely related species is difficult (Moss et al. 2020).

Examples of samples for which MAG recovery is challenging include those with a diverse array of microbes that do not yield sufficient reads for individual microbes, samples with many dissimilar microbes, and samples with large amounts of DNA from the host or external environment. Metagenomic sequences are typically obtained by sequencing randomly sampled DNA fragments, and consensus sequences are generated by integrating the fragmented sequences through de novo assembly. It is important to note that MAG itself only shows representative population consensus sequences of microbial genomes of the same species and genus and does not necessarily provide genomic information about individual bacterial strains (Van Rossum et al. 2020). Meanwhile, current metagenomic experiments are relatively easy to perform using commercial kits and public tools and are suitable for large sample analysis. Ideally, when DNA is extracted from environmental microbes with minimal fragmentation and read on a long-read sequencer, the MAG that is close to the complete genome can be obtained. Thus, metagenomics is a powerful analytical approach when sufficient microbial DNA samples are available or when microbial diversity is relatively simple.

In single-cell genomics, individual microbial cells are selectively or randomly isolated from a population, lysed, and their genome amplified using whole genome amplification (WGA) techniques. The genomic information obtained from these single cells is then sequenced, producing SAGs that are theoretically free from contamination or admixture with other organisms. This approach offers several advantages, such as the ability to link bacterial core genes in their genomes to exogenous mobile genetic factors such as plasmids and phages, the recovery of conserved genes such as 16S rRNA genes, and other genes often missing in conventional MAGs (Arikawa et al. 2021; Ide et al. 2022b). SAGs are collected individually, so their data quality is not affected by sample diversity or the presence of closely related microbes. Single-cell genomics is particularly useful for decoding individual genomes from highly diverse microbial samples or rare target microbes, which is difficult in the metagenomic binning approach. Examples of its application include the analysis of bacteria visible to the naked eye (Volland et al. 2022), a comprehensive survey of marine bacteria in surface seawater (Pachiadaki et al. 2019), the identification of secondary metabolite producers from marine sponges (Wilson et al. 2014; Kogawa et al. 2022), the assessment of subspecies and intraspecific recombination in environmental bacterial species (Zaremba-Niedzwiedzka et al. 2013; Kashtan et al. 2014), and the identification of gut bacteria that degrade soluble dietary fiber (Chijiiwa et al. 2020). Single-cell genomics provides previously inaccessible insights into microbial ecosystems and functions.

WGA is a critical step in generating sufficient amounts of DNA for subsequent genome sequencing. WGA methods include PCR-based methods, isothermal chain displacement reaction-based methods, and hybrid methods of both (de Bourcy et al. 2014; Gonzalez-Pena et al. 2021; Sobol and Kaster 2023), but the most widely used is the isothermal DNA amplification method with multiple displacement amplification (MDA) (Dean et al. 2001; Nishikawa et al. 2015). MDA amplifies DNA using phi29 polymerase and is characterized by its low error rate due to its exonuclease proofreading activity. Some improvements have been attempted to increase the efficiency of single-cell genome amplification by using thermostable phi29 and post-amplification treatment (Zhang et al. 2006; Stepanauskas et al. 2017). However, MDA-based amplified genomes often contain chimeric sequences, amplification products from contaminating DNA during the experimental process, and amplification bias. These factors prevent the assembly of contiguous sequences and result in a large number of short, fragmented contigs. In addition, low-amplified regions created by amplification bias are prevented from being sequenced, creating gaps in the SAG. Therefore, despite some potential advantages of SAGs over MAGs, most SAGs are of low quality, and the genomic completeness of SAGs averages only 40% or less of medium quality (Rinke et al. 2014).

## Technology to improve SAG acquisition efficiency

The success of single-cell genomics is highly dependent on the efficiency of cell isolation to prevent contamination and to provide a suitable environment for massively parallel reactions. In recent technologies, single cells are often encapsulated in droplets generated by microfluidic devices for high throughput analysis. Single-cell analysis microfluidic systems for gene expression of mammalian cells are commercially available and have become an essential tool for single-cell analysis researches (Svensson et al. 2018). Meanwhile, we have developed methods for single-cell genomics of microbes using microfluidic devices (Nishikawa et al. 2015; Hosokawa et al. 2017; Chijiiwa et al. 2020).

**Fig. 2:**
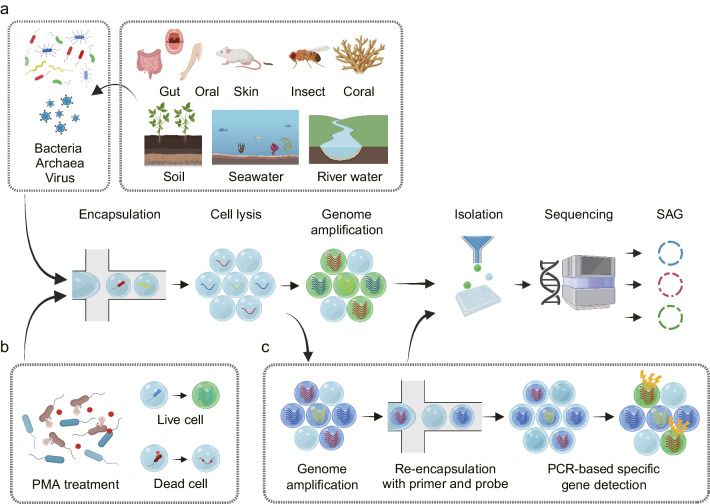
Schematic illustration of single-cell amplified genomes in gel beads sequencing (SAG-gel) and its applications. **a** The basic workflow of SAG-gel. The SAG-gel system is adaptable to bacteria, archaea, and viruses fractionated from various types of microbial samples. **b** PMA-SAG-gel obtains genomic information from viable cells by preventing genome amplification from dead cells. **c** Targeted gene detection by PCR after whole genome amplification helps the selection of amplified genomes of target cells

In single-cell amplified genomes in gel beads sequencing (SAG-gel), a comprehensive technique for microbial single-cell genomics developed by the authors (Chijiiwa et al. 2020; Nishikawa et al. 2022), massively parallel single-cell whole genome amplification is performed using picoliter volume droplets (Fig. 2a). Droplets are rapidly generated by shearing an agarose solution with carrier oil in a microfluidic channel. The agarose solution is pre-suspended with microbes diluted to less than one cell per droplet. The droplets are then collected in tubes and cooled to generate gel beads with a particle size of approximately 30 microns and trapping the microbes within the gel matrix. When the gel bead is immersed in a reagent, the trapped microbes are exposed to the reagent. Taking advantage of this property, 10^4^ to 10^6^ gel beads can be collectively immersed in multiple reagents sequentially. In addition, because the gel beads can be collected and washed by centrifugation, multiple treatments can be combined while removing reagent components that inhibit the next reaction. This feature allows a series of reactions from microbe lysis to whole genome amplification to be performed continuously within a single tube, which is expected to improve cell lysis efficiency. The gel capsule is an excellent environment for handling trace amounts of nucleic acids, as DNA can be purified by multi-step lysis in the gel and then transferred to genome amplification. This improves sequence quality even for Gram-positive bacteria, which are difficult to lyse and present challenges for obtaining data with conventional lysis methods (Nishikawa et al. 2022). The gel capsule can also be aliquoted into a multi-well plate using a cell sorter for long-term storage, and DNA indexes are attached to the amplified products and sequenced to obtain multiple SAGs comprehensively.

The advantage of SAG-gel over conventional single-cell genome sequencing techniques is the ability to obtain a large number of SAGs with superior completeness. Using this technology, a wide variety of SAGs have been obtained, including those from human commensals (intestinal (Chijiiwa et al. 2020; Hosokawa et al. 2022; Kogawa et al. 2023), oral, and skin-associated (Arikawa et al. 2021; Ide et al. 2022b)), coral/marine sponge commensals (Kogawa et al. 2022; Ide et al. 2022a), insect commensals (Arai et al. 2023), soil/rhizosphere bacteria (Yoda et al. 2020; Aoki et al. 2022), and river/marine bacteria. SAG-gel yields individual unknown microbial genomes even from samples containing a wide variety of microbes (including bacteria, archaea, and viruses) that are difficult to segregate by metagenomic analysis. This accumulation makes it possible to unravel the relationship between genes and genomes and to identify “which microbe is responsible for which function.” Even in samples with relatively low microbial diversity and a high degree of similarity, such as human commensal bacteria, it is possible to identify the genome of each bacterial strain and clarify the differences between similar but different bacterial strains. However, it should be noted that SAG-gel is not currently compatible with all microbes. Microbes that cannot be encapsulated in a gel bead due to their cell sizes or shapes, such as filamentous actinomycetes and fungi, cannot be analyzed. Microbes with high GC% tend to have low genomic recovery due to the characteristics of genome amplification bias. In addition, genome recovery tends to be lower for microbes that are difficult to lyse enzymatically because cells in the gel beads cannot undergo a physical lysis process (Chijiiwa et al. 2020; Nishikawa et al. 2022). Therefore, it is important to combine our technology with conventional microbial isolation and metagenomic analysis.

## Bioinformatic tools for improving SAG quality

**Fig. 3:**
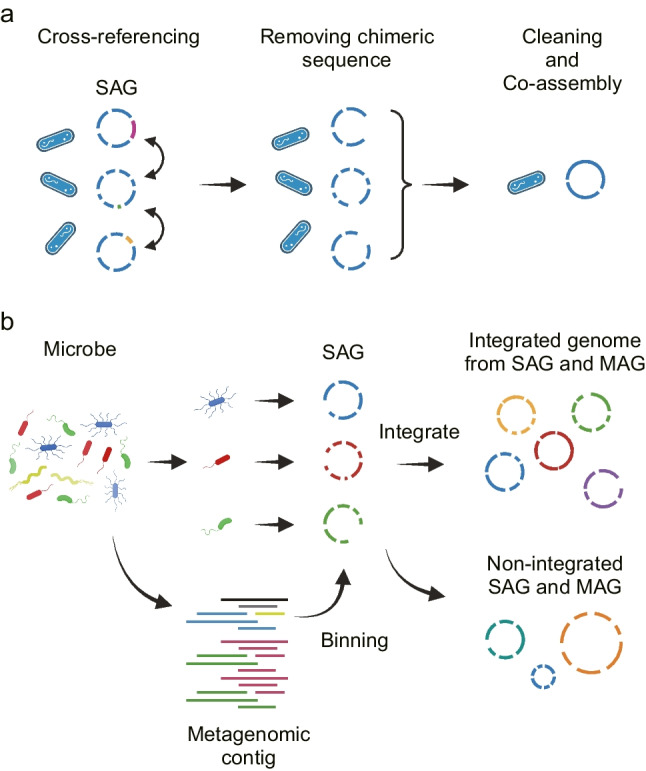
Bioinformatic tools for improving SAG quality. **a** Cleaning and co-assembly of the single-amplified genome (ccSAG) integrates multiple SAGs *in silico* while removing chimeric sequences. **b** SMAGLinker links sequencing data from single-cell genomics and metagenomics for the efficient construction of draft genomes

Since MDA exponentially amply the tiny amount of single-cell DNA, contaminating DNA, generation of chimeric reads, and amplification bias are the main causes which decrease the quality of SAGs. To address the issue of the accuracy of SAGs, we have also developed bioinformatics analysis tools. One such tool is ccSAG (cleaning and co-assembly of the single-amplified genome) (Kogawa et al. 2018), a method for removing chimeric sequences (Lasken and Stockwell 2007; Kiguchi et al. 2021), which are a unique problem in single-cell whole genome amplification and subsequent genome sequencing (Fig. 3a). This method involves comparing and integrating multiple SAGs that are presumed to be from the same species or strains *in silico* and identifying and eliminating sequences that do not overlap or are mapped to multi-distant loci. SAGs that are presumed to be from the same species are determined based on whole genome average nucleotide identity (ANI) and homology of marker genes. Since error sequences are detected by comparing SAGs, this method can be applied to data from uncultivated microbes for which no reference is available. The removal of chimeric sequences results in the elimination of sequence gaps and the integration of long contig sequences. In evaluations using *E. coli*, integrated six or more *E. coli* SAGs resulted in the same quality as those obtained by conventional sequencing of the purified extracted DNA. When multiple SAGs of low completeness have been obtained, ccSAG is an effective method for creating virtually integrated genomes with improved completeness of genomes.

The next analysis tool developed is SMAGLinker (Arikawa et al. 2021), which leverages the strengths of both single-cell genomics and metagenomics methods to construct draft genomes (Fig. 3b). This method performs metagenomic sequencing and single-cell genome sequencing on the same microbial sample as extracted DNA and suspended microbes, respectively. Then, the metagenomic contigs are paired using the SAG as a reference. The paired SAGs and metagenomic contigs are integrated using the one with higher completeness as a scaffold, thus improving genomic completeness. Unpaired SAGs or metagenomic contigs are output as standalone SAGs or MAGs. This approach combines the strengths of MAGs, which have comprehensive genomic information for microbial populations, with those of SAGs, which have high specificity for individual microbes, resulting in draft genomes that take advantage of all available genome sequencing data.

The effectiveness of SMAGLinker was evaluated using simulated samples containing 15 bacterial species, fecal-derived enterobacteria, and skin commensal bacteria (Arikawa et al. 2021). The results showed that SMAGLinker performed a more accurate sequence assignment than the metagenomics-alone approach and produced numerous high-quality bacterial genomes with high completeness in all samples. In contrast, the metagenomics-alone approach of skin microbial sequencing yielded an MAG for *Staphylococcus hominis* that was contaminated with many gene sequences from other bacterial species and had a genome size far from the estimated value, suggesting that it contained many mis-binned sequences. On the other hand, from the same skin swab sample, SMAGLinker yielded two appropriately sized staphylococcal strain genomes, each with a unique plasmid, indicating that they have different properties. SMAGLinker can be used to correct the failure of a metagenomics or single-cell genomics analysis to produce the expected result or when the result is questionable. In addition, the quality and number of MAGs or SAGs can be improved by obtaining additional data to perform SMAGLinker, even from samples that have already been analyzed.

## Methods for performing single-cell genome sequencing with specific targets

In most single-cell genomics research, bacteria and other microbes are randomly isolated to obtain SAGs. This random selection generally reflects the proportion of microbes present in the sample and can indicate the abundance of each microbe in the microbial ecosystem. However, since microbial research often focuses on microbes with specific functions or genes in microbial communities, there is a need for techniques that allow selective and detailed analysis of specific microbes. Here, we present three selective methods for single-cell genomics that we have developed.

The first approach is PMA-SAG-gel (Hosokawa et al. 2022), a technique for the specific analysis of the genome of living bacteria. Propidium monoazide (PMA) binds selectively to DNA in membrane-permeable dead cells and inhibits DNA amplification. By treating samples with PMA prior to introduction into the SAG-gel, single-cell genome sequencing can be performed in a viable cell-specific manner (Fig. 2b). PMA-SAG-gel was applied to human fecal samples and revealed the presence of bacterial strains with different viability, providing insight into species- and strain-level survival profiles in microbial populations. This technique will provide us with useful information for characterizing viable bacteria in specific environments, evaluating sample preparation conditions, and providing insight into the quality assessment of viable bacterial preparations.

The second approach we have developed is the use of specific sequences, such as the 16S rRNA gene, to selectively detect the amplified genomes of target microbes (Fig. 2c) (Ide et al. 2022a). In this method, gel beads containing the amplified genomes are suspended in a PCR mixture containing target-specific primers and probes, and the droplets are generated again. Then, PCR-based gene detection then allows selective genome sequencing of gel beads containing the target sequence. We have applied this targeted single-cell genomics method to coral commensal bacteria (Ide et al. 2022a) and insect symbiotic bacteria (Arai et al. 2023) and obtained target bacterial genome sequences that were difficult to obtain by conventional metagenomics and single-cell genomics. This method can selectively capture SAGs even when the presence ratio of target bacteria is approximately 1%, resulting in a 50-fold improvement in sequencing efficiency over random sampling and a significant reduction in reagents and labor. The third approach uses long-read DNA sequencing with a single-cell amplified genome long-read assembly (scALA) workflow (Kogawa et al. 2023). In this workflow, after the DNA is amplified by SAG-gel, the gel beads are randomly sorted, and sequence reads are obtained using a short-read sequencer. Then, by focusing on the qualified SAGs for the target species, long-read sequencing is also performed on the remaining amplified DNA. By using both short-read and long-read sequencers for selected SAGs, highly accurate genomic information can be obtained. In the application for human gut microbes, circular closed genomes were obtained using this workflow.

In conclusion, the experimental workflows presented here provide practical and comprehensive approaches for obtaining uncultured microbial genomes. These new techniques overcome the challenges of microbial single-cell genomics and can be used for highly accurate and specific collection of environmental microbial genomes. They improve the performance of single-cell genomics, such as throughput, accuracy, and selectivity, and provide genomic information with a high degree of confidence. The use of this technology enables a deeper understanding of the phylogenetic and functional details of target bacteria at the strain level, thereby extending the knowledge gained from conventional metagenomics approaches in microbiome research. Although most of the targets of single-cell genomics of microbes have been bacteria, similar techniques have been used to obtain genomes from archaea (Aoki et al. 2022), fungi (Ahrendt et al. 2018), and microbial eukaryotes (Ciobanu et al. 2021; Gollnisch et al. 2023) and have also been applied to single-particle genome analysis of viruses (Allen et al. 2011; Martinez-Hernandez et al. 2017). There are some challenges left for single-cell genomics of these microbes, including relatively large genome size and fragmented genomes; the application range of single-cell genomics is broadening. To date, microbiome research has focused mainly on discussions of the composition and balance of the microbial flora and functional annotations based on known reference genomes of related species, but this technology has the potential to open a new avenue for microbiome research by obtaining strain-resolved microbial genomes. We anticipate that microbial single-cell genomics will be widely used in medical research to unravel the relationship between various diseases and the human microbiome and in basic research to understand microbial evolution and its roles in the environment.

## Data Availability

Not applicable.
